# Numerical Analysis of Etoposide Induced DNA Breaks

**DOI:** 10.1371/journal.pone.0005859

**Published:** 2009-06-10

**Authors:** Aida Muslimović, Susanne Nyström, Yue Gao, Ola Hammarsten

**Affiliations:** Institute of Biomedicine, Department of Clinical Chemistry and Transfusion Medicine, The Sahlgrenska Academy at University of Gothenburg, Gothenburg, Sweden; Duke University United States of America.

## Abstract

**Background:**

Etoposide is a cancer drug that induces strand breaks in cellular DNA by inhibiting topoisomerase II (topoII) religation of cleaved DNA molecules. Although DNA cleavage by topoisomerase II always produces topoisomerase II-linked DNA double-strand breaks (DSBs), the action of etoposide also results in single-strand breaks (SSBs), since religation of the two strands are independently inhibited by etoposide. In addition, recent studies indicate that topoisomerase II-linked DSBs remain undetected unless topoisomerase II is removed to produce free DSBs.

**Methodology/Principal Findings:**

To examine etoposide-induced DNA damage in more detail we compared the relative amount of SSBs and DSBs, survival and H2AX phosphorylation in cells treated with etoposide or calicheamicin, a drug that produces free DSBs and SSBs. With this combination of methods we found that only 3% of the DNA strand breaks induced by etoposide were DSBs. By comparing the level of DSBs, H2AX phosphorylation and toxicity induced by etoposide and calicheamicin, we found that only 10% of etoposide-induced DSBs resulted in histone H2AX phosphorylation and toxicity. There was a close match between toxicity and histone H2AX phosphorylation for calicheamicin and etoposide suggesting that the few etoposide-induced DSBs that activated H2AX phosphorylation were responsible for toxicity.

**Conclusions/Significance:**

These results show that only 0.3% of all strand breaks produced by etoposide activate H2AX phosphorylation and suggests that over 99% of the etoposide induced DNA damage does not contribute to its toxicity.

## Introduction

Cancer is often treated with agents that induce DNA double-strand breaks (DSBs) that preferentially kill dividing cells and, therefore, are slightly more toxic to fast-growing tumor cells. The single-strand breaks (SSBs) that are always introduced along with the DSBs contribute little to the toxicity [1], [2]. DSBs activate several related and partially redundant protein kinases, including ATM, ATR and DNA-PK [3]. An early event after introduction of DSBs, but not other types of DNA damage, is the phosphorylation of a special form of histone 2A (H2A) denoted H2AX [4]. H2AX differs from its homologue H2A in that it contains a distinct C-terminal extension, with a consensus target sequence at serine 139 for the DSB-activated kinases ATM, ATR, and DNA-PK [4], [5]. Together, these kinases are responsible for the formation of several thousands of phosphorylated H2AX surrounding the DSB [5], [6], [7], [8]. This phosphorylation initiates the assembly of several proteins involved in the DSB response [9] and therefore mouse cells deleted for H2AX show several DSB-response defects [10], [11], [12], [13]. This, and several other lines of evidence, indicates that H2AX phosphorylation is required for the proper amplification of the DSB response [10]. The level of H2AX phosphorylation correlates closely with the level of DSBs and with the level of cell death in response to DSB-inducing agents such as ionizing radiation [14], [15], [16]. One of the most important DSB-inducing drugs in cancer treatment is etoposide. Etoposide induces DNA breaks by inhibition of topoisomerase II (topoII) [17], an enzyme that induces transient DSBs as part of its enzymatic mechanism [18], [19], [20], [21]. TopoII is a homodimer, of which each monomer is able to cleave and religate one DNA strand [22]. The cleavage reaction is mediated through a reactive tyrosine in the catalytic site that becomes covalently linked by a phosphotyrosyl-bond to the 5′-phosphate of the break [23]. The coordinated actions of each monomer result in efficient introduction of a topoII-linked DSB. After passage of an undamaged DNA molecule through the break, topoII religates the break and dissociates from DNA [24]. TopoII poisons such as etoposide specifically inhibit the religation step of the enzymatic cycle, and thereby locks covalently linked topoII to DNA [25]. Although topoII always induces DSBs when it cleaves DNA, etoposide is also capable of generating SSBs [22], [26], [27]. It has been found that etoposide must be bound to each monomer to prevent topoII from religating the break which leads to formation of the DSB. If only one monomer is bound by etoposide, the unbound topoII monomer reseals its break, generating a topoII-linked SSB [22]. Several lines of evidence indicate that most of the topoII-linked DSBs are repaired by religation of the breaks by the enzyme itself once etoposide has dissociated. However, if the TopoII-linked DSBs are encountered by an RNA or DNA polymerase, TopoII-DNA complex will be denatured [28], [29]. This likely renders topoII unable to religate the break and transforms the transient TopoII-linked DSBs into permanent DSBs. Detection of these denatured topoII-linked breaks likely involves removal of the denatured enzyme from the break. Several mechanisms have been proposed for this process including proteasome degradation [30], [31], [32] endonucleolytic processing [33] or tyrosyl-DNA phosphodiesterase mediated cleavage of the phosphotyrosyl bond [34], [35]. How the breaks are repaired is still unclear but, Ku and ligase IV are likely involved, since cells deficient in these functions are very sensitive to etoposide [36], [37], [38].

To examine etoposide-induced DNA damage further we have compared the effect of etoposide with that of calicheamicin (CLM), a drug that induces free (not protein-linked) DSBs. CLM binds to the minor groove in the DNA and induces DSBs by two radical centers present in the molecule. Generated DSBs mostly consist of a DNA strand ending with an abasic site and a 3′-phosphoglycolate on the other strand [39]. We have previously shown that 30% of CLM-induced DNA damage is DSBs that efficiently activate DNA-PK, ATM and H2AX phosphorylation [40], [41], [42]. In the present study we used a combination of methods to compare SSBs, DSBs, toxicity and H2AX phosphorylation induced by etoposide and CLM. CLM produces free DSBs and therefore serves as a control for the extent of processing of the topoII-linked DSBs to free DSBs. With this combination of methods we found that etoposide mostly induces SSBs, and that only a subset of the DSBs activates a DNA-damage response and cell death. These results indicate that most of the DNA damage induced by etoposide does not contribute to its toxicity.

## Results

### Etoposide induces mainly single-stranded DNA breaks

**Figure 1:**
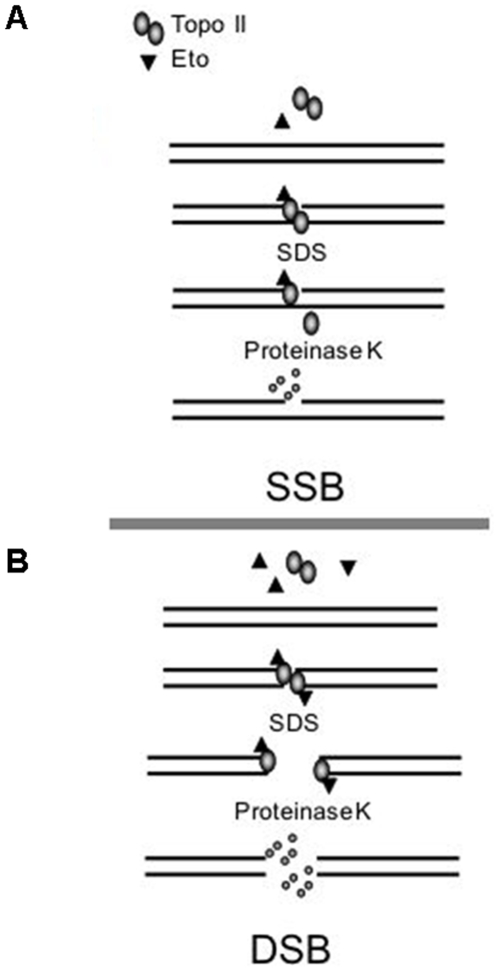
Etoposide-induced DNA breaks detected by CFGE. A homodimer of topoII binds and cleaves cellular DNA. Etoposide binds independently to each monomer to block religation and thereby locks the topoII-DNA complex. At low concentrations, only one of the topoII monomers will be bound by etoposide and unable to religate the break, resulting in a topoII-linked SSB (a). When both monomers are occupied by etoposide, a topoII-linked DSB will be generated (b). In CFGE cells are lysed and proteins removed from DNA by SDS and proteinase K, allowing detection of protein-linked SSBs and DSBs.

**Figure 2:**
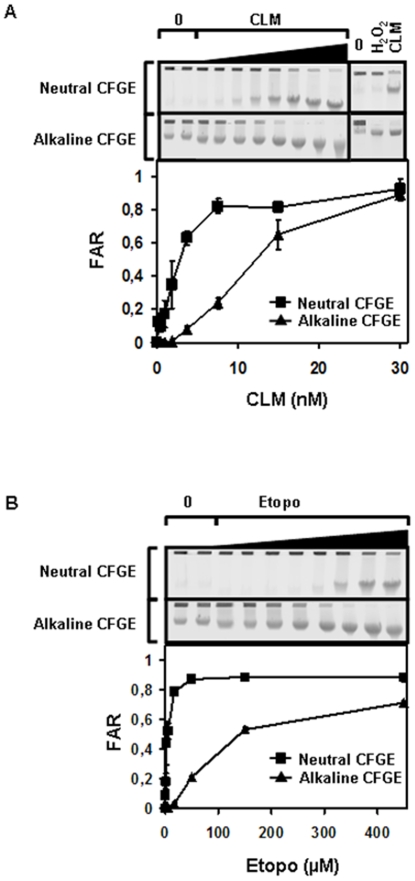
Strand breaks induced by etoposide and CLM. SV40-transformed fibroblasts treated with (a) CLM (0–30 nM) or (b), etoposide (0–450 µM) for 40 min at 37°C. The induced levels of TSBs and DSBs were measured with neutral and alkaline CFGE. As a control, we also treated cells with the SSB-inducer H_2_O_2_ (200 µM) or DSB and SSB inducer CLM (15 nM) to demonstrate that neutral CFGE fails to detect SSBs (a, separate gel). Error bars represent variation in two separate experiments performed on two different days.

To examine the relative amounts of SSBs and DSBs produced by etoposide (Fig. 1), we measured DNA strand breaks in cells treated with etoposide. As a control we also measured DNA strand breaks in cells treated with CLM or ionizing radiation (IR). A treatment time of 40 min was used in this and following experiments to facilitate comparisons between experiments. Measurements were done with a combination of neutral constant field gel electrophoresis (neutral CFGE) [43], [44], [45] and alkaline CFGE methods (Susanne Nyström, Louise Fornander, manuscript in preparation), which measure DSBs and total strand breaks, (TSBs) respectively, (TSB = SSB+DSB). This pair of methods therefore allows us to measure both DSBs and TSBs in a cell population. In these methods, cells are molded into agarose plugs and subjected to electrophoresis after in-gel lysis by SDS and proteinase K. If the cells contain DNA breaks, ds DNA fragments smaller than 10 Mbp migrate into the agarose gel. Since most of the chromatin proteins are removed from DNA, CFGE measures all topoII induced DNA breaks irrespective of cellular processing of the covalently attached topoII (Fig. 1). The fraction of the DNA that enters the gel (FAR) is proportional to the number of DNA strand breaks. If the electrophoresis is performed under neutral conditions (neutral CFGE) the method measures DSBs, whereas under alkaline conditions (alkaline CFGE), the method measures both DSBs and SSBs (TSBs). To show that alkaline CFGE and neutral CFGE were able to distinguish between DSBs and SSBs, we treated cells with H_2_O_2_ that produces predominantly SSBs at these concentrations [42]. As expected, H_2_O_2_, which induces very few DSBs [42] produced FAR only in alkaline CFGE, whereas no increased FAR signal was obtained in neutral CFGE (Fig. 2A). In contrast, CLM-treated cells produced FAR under both neutral and alkaline conditions reflecting that CLM induces both SSBs and DSBs (Fig. 2A)

From this analysis, it was evident that etoposide predominantly induced SSBs, since alkaline CFGE detected DNA strand breaks at etoposide concentrations that did not result in any detectable levels of DSBs by neutral CFGE (Fig. 2B). In CLM-treated cells, the ratio between DSBs and SSBs was higher indicating that CLM induced more DSBs per SSB (Fig. 2A), as expected from our previous work. To calculate the relative amount of SSBs and DSBs produced by etoposide and CLM, we also measured TSBs and DSBs in cells irradiated with IR (data not shown). We then compared the FAR values from irradiated cells with the FAR values obtained in etoposide- or CLM-treated cells. It is known that 4% of IR-induced strand breaks are DSBs and 96% are SSBs [1]. By comparing the FAR values from the IR-irradiated and etoposide-treated cells, we could calculate that 1 µM etoposide induced TSBs to the same level as 7 Gy, and DSBs to the same level as 5 Gy. Since 1 Gy of IR produces 40 DSBs/cell and 1040 TSBs/cell [1], this comparison shows that 1 µM of etoposide induces 200 DSBs/cell and 7280 TSB/cell. We could therefore calculate that 97% of all strand breaks produced by etoposide were SSBs, and less than 3% of the strand breaks were DSBs. In CLM-treated cells, DSBs constituted 31% and SSBs 69% of all strand breaks, as we have previously shown [41]. In conclusion, we find that etoposide induces 30-fold more SSBs than DSBs.

### Etoposide-induced DSBs are inefficient inducers of H2AX phosphorylation

To examine to what extent etoposide induced DSBs activate H2AX phosphorylation, we compared the levels of DSBs, measured by neutral CFGE, and H2AX phosphorylation in cells treated with etoposide or CLM. Neutral CFGE measures all topoII induced DSBs irrespective of cellular processing of the covalently attached topoII (Fig. 1B), while H2AX phosphorylation only reflects DSBs that are detected by cellular DNA-damage response systems. A treatment time of 40 min was used, since this results in a maximal induction of DSBs by both drugs. CLM produces free DSBs and it is therefore expected that all CLM-induced DSBs will induce H2AX phosphorylation. CLM therefore serves as a control for the extent of processing of the topoII-linked DSBs to free DSBs.

**Figure 3:**
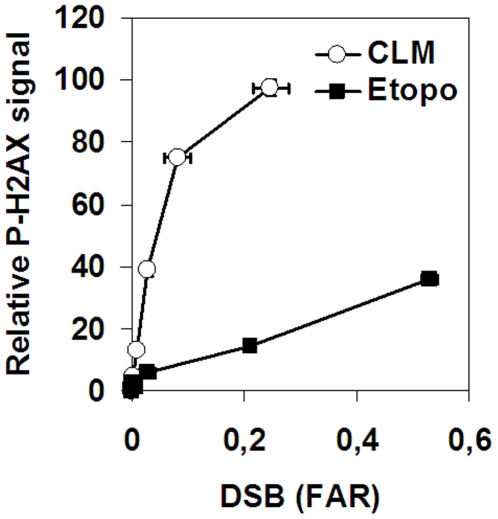
Induction of H2AX phosphorylation by etoposide- or CLM-induced DSBs. SV40-transformed fibroblasts were treated with 0–150 µM etoposide or 0–5 nM CLM before analysis of H2AX phosphorylation and DSB-level by neutral CFGE. Error bars represent variation in two separate experiments performed on different days.

When we plotted DSB formation against H2AX phosphorylation, it was evident that CLM induced a 20 fold higher level of H2AX phosphorylation compared to etoposide (Fig. 3, Supplementary figures S1 and S2). For instance, when we compare levels of DSBs induced by CLM and etoposide at similar H2AX phosphorylation levels (38 and 37 respectively), we find that CLM induces a FAR value of 0.02 and etoposide induces FAR value of 0.53 (Fig. 3, Supplementary figures S1 and S2). Assuming that all CLM-induced DSBs activated H2AX phosphorylation, our data indicates that only 1 out of 20 (5%) etoposide-induced DSBs activate H2AX phosphorylation.

### Etoposide-induced DSBs are 10-fold less toxic than CLM-induced DSBs

**Figure 4:**
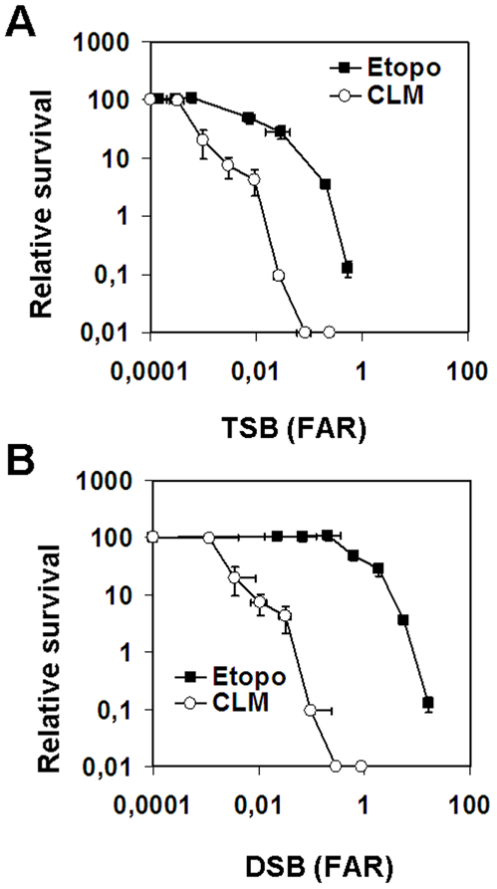
Effect on cell survival of etoposide- and CLM-induced DSBs and TSBs. SV40-transformed fibroblasts were treated with 0–150 µM etoposide or 0–5 nM CLM for 40 minutes at 37°C before analysis of colony survival and levels of (a) TSBs or (b) DSBs by neutral and alkaline CFGEs calculated as described in materials and methods. Error bars represent variation in two separate experiments performed on two different days.

We also wanted to examine the biological importance of the strand breaks induced by etoposide. We therefore analyzed colony survival of the cells used in figure 3. When TSBs measured by alkaline CFGE were plotted against survival, it was evident that CLM-induced TSBs were close to 100-fold more toxic than etoposide-induced TSBs (Fig 4A). Part of this difference could be due to the fact that CLM induces 10-fold more DSBs than etoposide (31% versus 3%). However, when DSBs were plotted against survival, the CLM-induced DSBs were still 10-fold more toxic than the etoposide-induced DSBs (Fig. 4B). We conclude that etoposide-induced DSBs are 10-fold less toxic than CLM-induced DSBs.

### The toxic effect of etoposide-induced DNA breaks correlates closely to the level of H2AX phosphorylation

**Figure 5:**
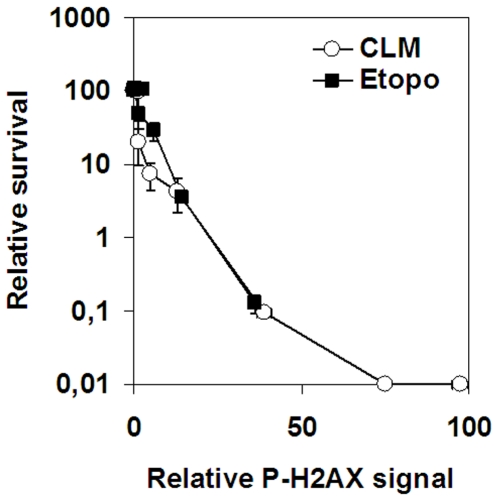
Cell survival and H2AX phosphorylation in response to etoposide or CLM. SV40-transformed fibroblasts were treated with 0–150 µM etoposide or 0–5 nM CLM before analysis of colony survival and H2AX phosphorylation. Error bars represent variation in two separate experiments performed on two different days.

To further explore the difference in cell death induced by etoposide and CLM, we plotted survival against the level of H2AX phosphorylation, which reflects DSBs that are detected by cellular DNA-damage response systems (Fig. 5).

The data show that the survival at any given level of H2AX phosphorylation was similar for etoposide and CLM. This opens the possibility that etoposide-induced cell killing is due to the few DSBs that induce H2AX phosphorylation.

### Etoposide induces DSBs and H2AX phosphorylation in a cell cycle-independent manner

**Figure 6:**
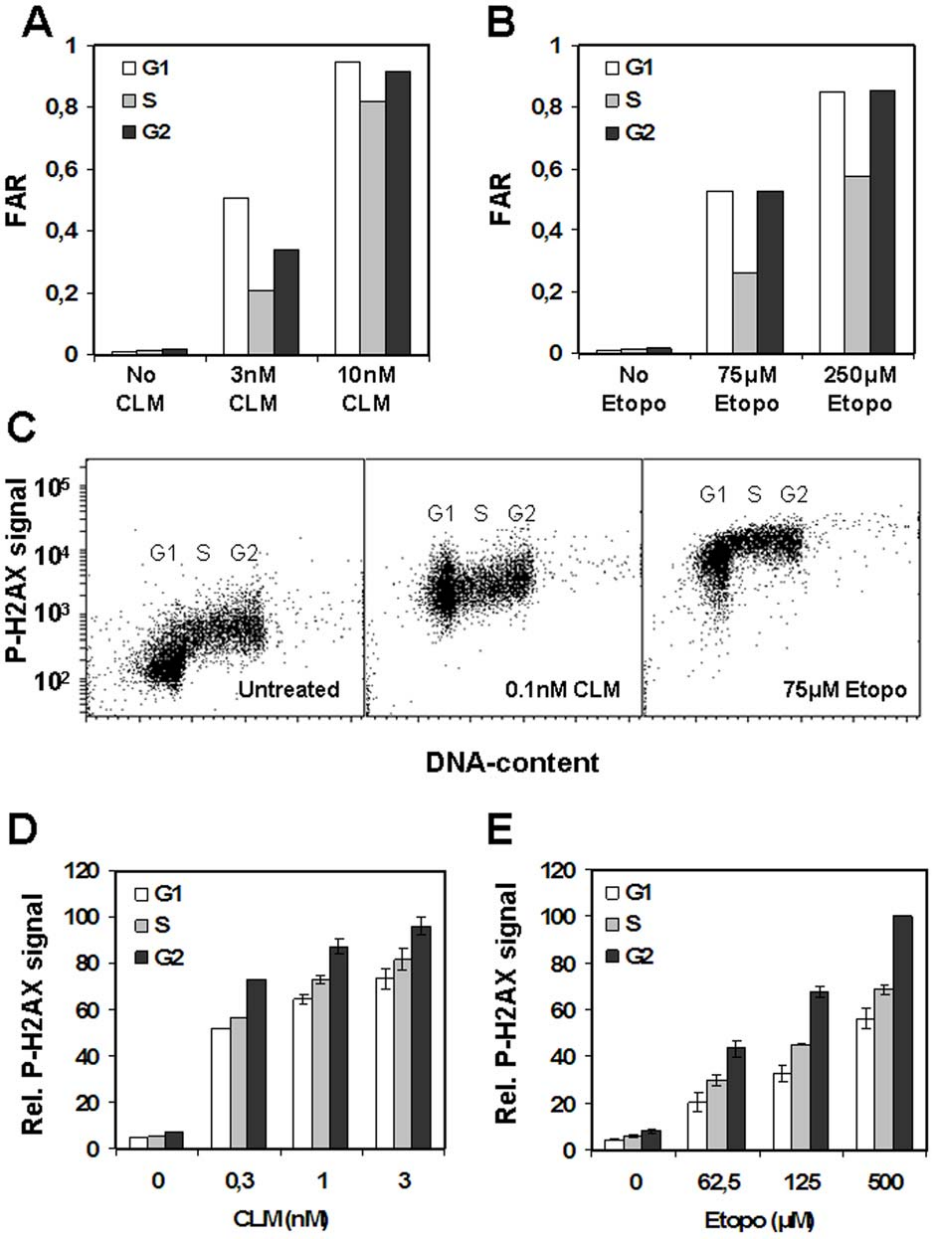
Induction of DSBs and H2AX phosphorylation at different cell-cycle stages. G361 cells were treated with 3 or 10 nM CLM (a) or 75 µM or 250 µM Etoposide (b) for 40 minutes before DNA staining, FACS sorting of G1, S and G2 cells, and analysis of DSBs by neutral CFGE. G361 cells were untreated or treated with 0.1 nM CLM or 75 µM Etoposide (c) for 40 minutes before DNA staining and analysis of H2AX phosphorylation and DNA-content to examine H2AX phosphorylation in G1, S and G2 cells. G361 cells were treated with CLM (d) or Etoposide (e) for 40 minutes before DNA staining and analysis of H2AX phosphorylation. Error bars represent variation from two separate experiments performed on two different days.

One potential difference between etoposide and CLM is that topoII poisons could induce more DNA strand breaks during the S-phase of the cell cycle. Topoisomerases are expected to be more active in S-phase, where they participate in relieving torsional stress ahead of the replication forks [18]. In addition, topoII alpha has been shown to be expressed at higher levels in dividing cells [19]. CLM, on the other hand, cleaves DNA by a radical-mediated process, and is not expected to induce DSBs in a cell cycle-dependent manner. To examine this further, we first sorted etoposide- or CLM-treated cells in G1, S, and G2 phases using FACS according to DNA content, and measured the level of DSBs by neutral CFGE and H2AX phosphorylation (Fig. 6). No apparent difference in the cell cycle distribution of DSBs or H2AX phosphorylation was evident in etoposide- or CLM-treated cells. It is therefore unlikely that the different toxicity elicited by etoposide- and CLM-induced DSBs was due to differences in the cell-cycle distribution of the breaks.

### Etoposide-induced DSBs induce H2AX phosphorylation with slow kinetics

Previous reports indicate that etoposide-induced DSBs must be denatured by RNA or DNA polymerases and the topoII moiety must also be removed in order to induce H2AX phosphorylation [30], [31], [32]. It is therefore expected that etoposide-induced DSBs would induce H2AX phosphorylation with a delay compared with CLM-induced DSBs.

**Figure 7:**
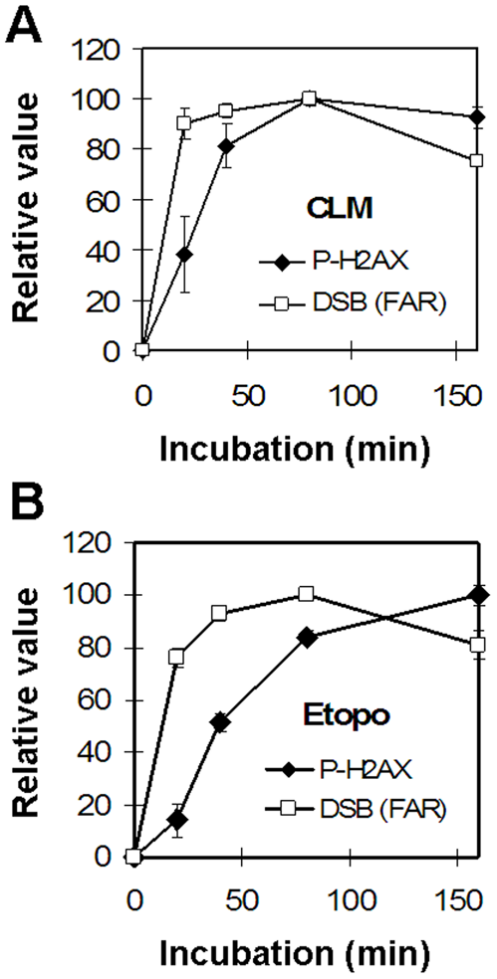
Time-dependent induction of DSBs and H2AX phosphorylation. Analysis of DSBs and H2AX phosphorylation in SV40-transformed fibroblasts treated with 3 nM CLM (a) or 250 µM etoposide (b) for 0, 20, 40, 80 or 160 minutes at 37°C before analysis of DSBs with neutral CFGE and H2AX phosphorylation. Error bars represent variation in two separate experiments performed on two different days.

To examine this possibility, we incubated cells with etoposide or CLM for different times and measured the accumulation of DSBs via neutral CFGE and H2AX phosphorylation (Fig. 7). In CLM-treated cells, DSBs reached a maximum after 20 min, resulting in maximal H2AX phosphorylation after 40 min (Fig. 7A). In etoposide-treated cells, the DSB induction was maximal after 40 min, but H2AX phosphorylation continued to accumulate and reached its maximum only after 160 min (Fig. 7B). This indicates that etoposide-induced DSBs require additional processing to induce H2AX phosphorylation. These data also show that after 40 min etoposide exposure, H2AX phosphorylation is close to 50% of its maximal value. Since we used a 40 min incubation for the experiments shown in figure 3, these data indicate that even after extended etoposide exposure, no more than 10% of all topoII-linked DSBs are converted to free DSBs that can activate H2AX phosphorylation.

## Discussion

Here we have compared DNA strand breaks induced by etoposide with the free strand breaks induced by CLM. We used a combination of methods to measure DSBs, SSBs, toxicity and H2AX phosphorylation to examine the relative amounts of strand-breaks, DNA damage signaling and cell survival. We found that only 3% of all DNA strand breaks induced by etoposide are DSBs. Previous reports have also indicated that etoposide mostly induces SSBs [26], [27], although, to the best of our knowledge, this is the first time that the relative amounts of SSBs and DSBs have been measured. It is therefore necessary to modify the prevailing paradigm that etoposide is a specific DSB-inducing agent. We found that etoposide-induced strand breaks were 100-fold less toxic than CLM-induced strand breaks. This 100-fold difference could be explained by a combination of the 10-fold lower DSB fraction induced by etoposide (34% for CLM versus 3% DSBs for etoposide) and the 10-fold lower levels of H2AX phosphorylation produced by etoposide induced DSBs. We also found that at the same level of DSBs, etoposide was 10-fold less toxic than CLM and produced 20-fold lower levels of H2AX phosphorylation. Our data indicate that a small fraction of all etoposide-induced DSBs activate H2AX phosphorylation, suggesting that only DSBs have been processed to free DSBs activate the cellular DNA-damage response system. By comparing the level of DSBs and H2AX phosphorylation in CLM- or etoposide-treated cells, we calculated that only 5% of all etoposide-induced DSBs induced H2AX phosphorylation during the 40-min exposure in this experiment. Our data also show that H2AX phosphorylation reaches 50% of its maximal level after 40 min. Therefore, even after extended exposure to etoposide at most 10% of the topoII-linked DSBs are converted to H2AX foci. The different toxicities of CLM- and etoposide-induced DNA breaks could not be attributed to preferential induction of DNA damage at different cell-cycle stages, since the distribution of H2AX phosphorylation was not cell-cycle dependent, in agreement with previous results [16], [46]. We also used neutral CFGE on FACS sorted cells to show that etoposide induces DSBs in a cell cycle independent manner.

Our data indicate that possibly as much as 90% of the DSBs produced by etoposide are held in a topoII-linked complex that is not recognized by ATM, DNA-PK, or other DNA-damage recognition systems. In line with this possibility, we have shown that purified DNA-PK fails to recognize topoII-linked DSBs [47]. A previous report shows that etoposide fail to activate PARP indicating that also the topoII-linked SSBs remain hidden from the cellular SSB detection system [48]. It is likely that most topoII-linked strand-breaks are simply religated by topoII when etoposide is removed and therefore not recognized by the cell.

**Figure 8:**
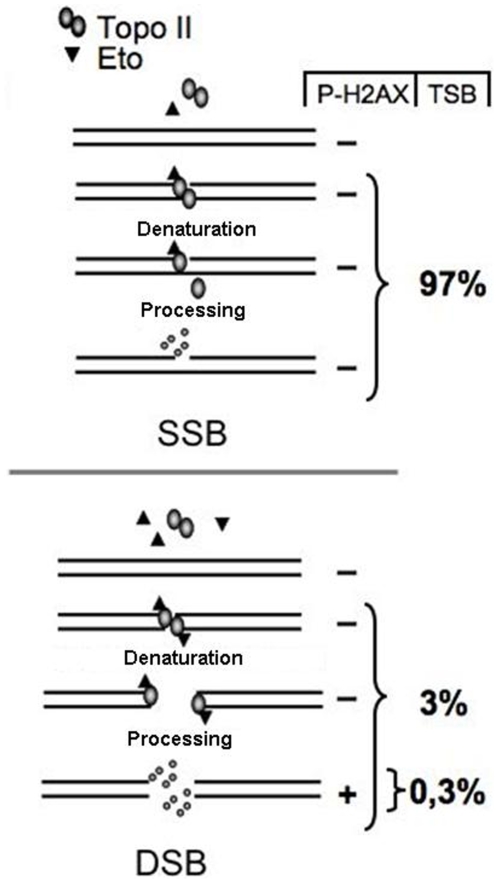
Etoposide-induced DNA damage in cells. A homodimer of topoII binds and cleaves cellular DNA, generating a topoII-linked DSB. Etoposide binds independently to each monomer to block religation, locking the topoII monomer to the DNA break. If only one of the topoII monomers is bound by etoposide and unable to religate the break, this results in a topoII-linked SSB (a). When both monomers are occupied by etoposide, a topoII-linked DSB will be stabilized (b). TopoII-linked DNA breaks that are encountered by RNA or DNA polymerases during etoposide exposure will be denatured and therefore unable to religate the breaks. Denatured topoII will be cleared from the breaks, resulting in free DSBs that can induce H2AX phosphorylation. The relative amounts of these breaks as a percentage of all etoposide-induced breaks are indicated.

Previous studies show that the etoposide-induced DNA-damage response is attenuated if cells are exposed to inhibitors of RNA and DNA synthesis [28], [29], [32]. It is therefore likely that etoposide-blocked topoII complexes become denatured and unable to religate the break only if they are encountered by an RNA- or DNA-polymerase (Fig. 8). Purified DNA-PK fails to recognize denatured topoII-linked breaks, partly because its DNA-binding subunit Ku is unable to bind [47]. The fact that proteasome inhibitors attenuate etoposide-induced H2AX phosphorylation indicates that removal of denatured topoII from DSBs involves proteasome-mediated degradation of topoII at the break [32]. It is also possible that removal of topoII occurs by other mechanisms, for instance by a nuclease similar to Spo11-removal during meiotic recombination [33] or by a specific cleavage of the phosphotyrosyl bond [34], [35]. Regardless of the mechanism, our data indicate that recognition and repair of denatured topoII breaks requires processing to produce free DSBs. Repair of these DSBs likely occurs in a process involving Ku and Ligase IV, since mutant cells show an extreme sensitivity to etoposide [36], [37], [38].

A relevant question is whether the topoII-induced SSBs contribute to the toxicity of etoposide. Etoposide could function similarly to camptothecin, which introduces topoisomeraseI-linked SSBs that can be converted to DSBs during DNA synthesis via replication fork collapse [28], [49]. Several reports show that camptothecin-induced DSBs, H2AX phosphorylation, and cell death can be prevented if cells are prevented from entering S-phase [49]. Our data suggest that this mechanism is likely not a major cause of etoposide-induced toxicity since we found that the levels of DSBs and H2AX phosphorylation were not increased in S-phase cells. It is therefore likely that etoposide confers its toxicity exclusively by the few free DSBs that it produces. In support of this interpretation, we found a close correlation between cell death and H2AX phosphorylation by etoposide and CLM. Our data therefore support the possibility that topoII-linked DSBs that become processed to free DSBs are responsible for etoposide induced toxicity.

## Materials and Methods

### Cells and reagents

Calicheamicin γ1 (CLM) was a generous gift from George Ellestad (Wyeth-Ayers Research). CLM was dissolved at 2 mM in DMSO and stored at −70°C. Etoposide (Sigma) was dissolved at 170 mM in DMSO and stored at −20°C. Simian virus-40 (SV-40)-transformed human fibroblasts (Coriell Institute for Medical Research) were cultured in DMEM (Invitrogen) supplemented with 10% fetal bovine serum, and penicillin/streptomycin. G361 human melanoma cells were grown in McCoys modified DMEM (Invitrogen). Cells were grown in a humidified atmosphere with 5% CO_2_.

### Measurement of DSBs and TSBs

DSBs were measured by neutral constant field gel electrophoresis (neutral CFGE) [43], [44], [45] and quantified as described for alkaline CFGE method. TSBs (DSBs+SSBs) were measured by alkaline CFGE method (Susanne Nyström, Louise Fornander manuscript in preparation). After drug exposure, the cells were kept on ice until scraping, and all solutions added to the cells were ice-cold. Cells treated with CLM were incubated on ice with PBS supplemented with 0.2 mg/ml sheared herring sperm DNA and 56 mM β-mercaptoethanol to inactivate excess CLM. The cells were scraped from the plates, centrifuged and washed with PBS. The washed cells were suspended in PBS and mixed with an equal volume of melted agarose (1.25% type VII in PBS with 5 mM EDTA) kept at 55°C and transferred to a plug mold (Biorad) and allowed to solidify on ice for at least 10 min. The gel plugs were then transferred to 330 µl of ice-cold deproteinization buffer (25 mM EDTA, pH 8.5, 0.5% SDS, 3 mg/ml proteinase K added just prior to deproteinization) and incubated at 4°C over night. Longer incubation times increased the FAR value in untreated cells likely because of induction of DNA strand breaks during incubation. The plugs were then equilibrated in 1 ml of 1 mM Tris-HCl, pH 7.5 for 1.5 h to remove SDS and proteinase K and subsequently equilibrated in alkaline buffer (0.03 M NaOH, 2 mM EDTA, 0.5 M NaCl, 0.05% BFB) for 1.5 h. The equilibrated gel plugs were then molded into a 0.7% agarose gel mixed in water the day before and equilibrated in alkaline buffer (0.03 M NaOH, 2 mM EDTA (pH 12.5)) over night at 4°C. The assembled alkaline gel was run at 4°C for 17 h at 0.6 V/cm in alkaline buffer (0.03 M NaOH, 2 mM EDTA (pH 12.5). The pH in the alkaline CFGE is set to allow DNA strand-separation of the smallest chromosomes, as this results in the highest sensitivity to detect TSBs. Since even chromosome-sized single-stranded DNA can migrate in agarose gels, denaturation of the smallest chromosomes results in a background corresponding to a FAR of 0.2 and does not reflect that untreated cells contain SSBs. The FAR from untreated cells were therefore subtracted when the FAR in drug treated cells were calculated. The alkaline conditions used in the alkaline CFGE degrade all RNA, omitting the need for an RNase step. Alkaline gel was run directly after molding of the gel plugs. If the gel was run the next day, NaCl in the gel plugs was lost by diffusion resulting in low FAR signal. The gels were stained by equilibration with stain buffer (25 mM Tris-HCl pH 7.5, µg/ml ethidium bromide) for at least 3 hours. Initially the CFGE was done using 3H-thymidine labeled cells, allowing quantification of the DNA by scintillation counting. We found that densitometric analysis of the CFGE gels after staining with ethidium bromide gave exactly the same results as quantification of the radioactivity in the gels. We therefore used densitometric quantification of the DNA in all subsequent experiments. The gels were scanned with a laser scanning equipment (Typhoon 9200 Variable Mode Imager). The relative amount of cellular DNA migrating into the gel, fraction of activity released, FAR was quantified using ImageQuant 5.2. Briefly, background subtracted signal in a rectangle covering the gel plug and the DNA that migrated into the gel was regarded as the “total DNA signal”. The background-subtracted signal from a smaller rectangle drawn 1 mm below the gel plug covering the DNA that entered the gel was regarded as the “activity released”. FAR was calculated by the following equation: FAR = activity released/total DNA signal. Relative amounts of DSBs and SSBs induced by etoposide were calculated by extrapolation from the linear part of the data set and compared to the FAR values from parallel experiments using IR. It is known that 1 Gy of ionizing radiation induces at most 40 DSBs and 1040 TSBs per cell [1]. The FAR value from the neutral CFGE and alkaline CFGE was used to find a drug concentration that produced a FAR value corresponding to 1 Gy. For etoposide it was found that 1 µM induced DSBs equal to 5 Gy and TSBs to a level corresponding to 7 Gy of IR. Using the formula: SSBs = TSBs−DSBs, we calculated that 1 µM of etoposide induced 200 DSBs/cell and 7280 SSBs/cell.

### Measurement of H2AX phosphorylation

H2AX phosphorylation was analyzed by (FACS) analysis as described [50], [51]. At least 100 000 cells in a 50 µl suspension (PBS, 1 g/l BSA) were added to 150 µl Block-9 staining buffer (PBS, 1 g/l BSA, 8% mouse serum, 0.1 g/l RNaseA, phosphatase inhibitors (10 mM NaF, 1 mM Na_2_MoO_4,_ 1 mM NaVO_3_), 0.25 g/l herring sperm DNA, 0.1% Triton X-100, 5 mM EDTA, 0.05% NaN_3_, mouse monoclonal anti-H2AXS139ph FITC conjugate (Millipore)) and stained in dark for 3 h at 4°C. Cell cycle distribution was monitored by addition of 5 µM Vybrant dye cycle violet stain (Invitrogen) during the last staining hour. The samples were then diluted with 300 µl suspension buffer (PBS, 1 g/l BSA) and analyzed. FACS analysis of cell cycle distribution and FACS sorting of G1, S, and G2 cells based on DNA content was done using FACSAria (B&D) with the following settings: 488 nm and 405 nm excitation lasers were used for excitation of FITC and Vybrant Dye Cycle Violet Stain respectively. Emission was detected with the filter/bandpass: 450/40 for FITC and 530/30 for Vybrant Dye Cycle Violet Stain. Fluorescence intensity in arbitrary units was plotted in histograms and the mean fluorescence intensity was calculated using Weasel version 2.3 software

### Colony survival

Cells were treated with CLM (0, 0.02, 0.06, 0.19, 0.55, 1.67 and 5 nM) or etoposide (0, 0.2, 0.6, 1.85, 5.6, 16.7, 50, 150 µM) for 40 minutes before washing and trypsinization. To allow direct comparison of the number of strand-breaks and H2AX phosphorylation, cells from the same experiment (Fig. 3, 4 and 5) were also analyzed by alkaline CFGE, neutral CFGE and H2AX phosphorylation. For the colony assay, cells were serially diluted and plated at different densities and grown for two weeks in normal growth medium to allow the colonies to expand. Cells were then fixed in methanol and stained with Giemsa before colony counting by eye. Smaller colonies were examined in a microscope. A colony was defined as a coherent assembly of more than 50 cells.

## Supporting Information

Figure S1 DNA strand break induction and H2AX phosphorylation by calicheamicin. SV40-transformed fibroblasts were treated with 0–5 nM CLM for 40 minutes before analysis of H2AX phosphorylation and survival using the colony assay. Data generated from different days are shown.
(0.07 MB TIF)

Figure S2 DNA strand break induction and H2AX phosphorylation by etoposide. SV40-transformed fibroblasts were treated with 0–150 µM etoposide for 40 minutes before analysis of H2AX phosphorylation and survival using the colony assay. Data generated from different days are shown.
(0.07 MB TIF)

## References

[1] Nias AHW (1998). An introduction to radiobiology, 2nd edition.

[2] Tounekti O, Kenani A, Foray N, Orlowski S, Mir LM (2000). The ratio of single- to double-strand DNA breaks and their absolute values determine cell death pathway.. Br J Cancer.

[3] Shiloh Y (2003). ATM and related protein kinases: safeguarding genome integrity.. Nat Rev Cancer.

[4] Rogakou EP, Pilch DR, Orr AH, Ivanova VS, Bonner WM (1998). DNA double-stranded breaks induce histone H2AX phosphorylation on serine 139.. J Biol Chem.

[5] Rogakou EP, Boon C, Redon C, Bonner WM (1999). Megabase chromatin domains involved in DNA double-strand breaks in vivo.. J Cell Biol.

[6] Burma S, Chen BP, Murphy M, Kurimasa A, Chen DJ (2002). ATM Phosphorylates Histone H2AX in Response to DNA Double-strand Breaks*.. J Biol Chem.

[7] Stiff T, O'Driscoll M, Rief N, Iwabuchi K, Lobrich M (2004). ATM and DNA-PK function redundantly to phosphorylate H2AX after exposure to ionizing radiation.. Cancer Res.

[8] Friesner JD, Liu B, Culligan K, Britt AB (2005). Ionizing radiation-dependent gamma-H2AX focus formation requires ataxia telangiectasia mutated and ataxia telangiectasia mutated and Rad3-related.. Mol Biol Cell.

[9] Stucki M, Jackson SP (2006). gammaH2AX and MDC1: anchoring the DNA-damage-response machinery to broken chromosomes.. DNA Repair (Amst).

[10] Celeste A, Petersen S, Romanienko PJ, Fernandez-Capetillo O, Chen HT (2002). Genomic instability in mice lacking histone H2AX.. Science.

[11] Bassing CH, Chua KF, Sekiguchi J, Suh H, Whitlow SR (2002). Increased ionizing radiation sensitivity and genomic instability in the absence of histone H2AX.. Proc Natl Acad Sci U S A.

[12] Bassing CH, Alt FW (2004). H2AX may function as an anchor to hold broken chromosomal DNA ends in close proximity.. Cell Cycle.

[13] Fernandez-Capetillo O, Lee A, Nussenzweig M, Nussenzweig A (2004). H2AX: the histone guardian of the genome.. DNA Repair (Amst).

[14] Olive PL, Banath JP (2004). Phosphorylation of histone H2AX as a measure of radiosensitivity.. Int J Radiat Oncol Biol Phys.

[15] MacPhail SH, Banath JP, Yu TY, Chu EH, Lambur H (2003). Expression of phosphorylated histone H2AX in cultured cell lines following exposure to X-rays.. Int J Radiat Biol.

[16] Banath JP, Olive PL (2003). Expression of phosphorylated histone H2AX as a surrogate of cell killing by drugs that create DNA double-strand breaks.. Cancer Res.

[17] Burden DA, Osheroff N (1998). Mechanism of action of eukaryotic topoisomerase II and drugs targeted to the enzyme.. Biochim Biophys Acta.

[18] Champoux JJ (2001). DNA topoisomerases: structure, function, and mechanism.. Annu Rev Biochem.

[19] Fortune JM, Osheroff N (2000). Topoisomerase II as a target for anticancer drugs: when enzymes stop being nice.. Prog Nucleic Acid Res Mol Biol.

[20] McClendon AK, Osheroff N (2007). DNA topoisomerase II, genotoxicity, and cancer.. Mutat Res.

[21] Li TK, Liu LF (2001). Tumor cell death induced by topoisomerase-targeting drugs.. Annu Rev Pharmacol Toxicol.

[22] Bromberg KD, Burgin AB, Osheroff N (2003). A two-drug model for etoposide action against human topoisomerase IIalpha.. J Biol Chem.

[23] Liu LF, Rowe TC, Yang L, Tewey KM, Chen GL (1983). Cleavage of DNA by mammalian DNA topoisomerase II.. J Biol Chem.

[24] Berger JM, Gamblin SJ, Harrison SC, Wang JC (1996). Structure and mechanism of DNA topoisomerase II.. Nature.

[25] Osheroff N (1989). Effect of antineoplastic agents on the DNA cleavage/religation reaction of eukaryotic topoisomerase II: inhibition of DNA religation by etoposide.. Biochemistry.

[26] Wozniak AJ, Ross WE (1983). DNA damage as a basis for 4′-demethylepipodophyllotoxin-9-(4,6-O-ethylidene-beta-D-glucopyranoside) (etoposide) cytotoxicity.. Cancer Res.

[27] Long BH, Musial ST, Brattain MG (1986). DNA breakage in human lung carcinoma cells and nuclei that are naturally sensitive or resistant to etoposide and teniposide.. Cancer Res.

[28] D'Arpa P, Beardmore C, Liu LF (1990). Involvement of nucleic acid synthesis in cell killing mechanisms of topoisomerase poisons.. Cancer Res.

[29] Bodley AL, Huang HC, Yu C, Liu LF (1993). Integration of simian virus 40 into cellular DNA occurs at or near topoisomerase II cleavage hot spots induced by VM-26 (teniposide).. Mol Cell Biol.

[30] Mao Y, Desai SD, Ting CY, Hwang J, Liu LF (2001). 26 S proteasome-mediated degradation of topoisomerase II cleavable complexes.. J Biol Chem.

[31] Desai SD, Li TK, Rodriguez-Bauman A, Rubin EH, Liu LF (2001). Ubiquitin/26S proteasome-mediated degradation of topoisomerase I as a resistance mechanism to camptothecin in tumor cells.. Cancer Res.

[32] Zhang A, Lyu YL, Lin CP, Zhou N, Azarova AM (2006). A protease pathway for the repair of topoisomerase II-DNA covalent complexes.. J Biol Chem.

[33] Neale MJ, Pan J, Keeney S (2005). Endonucleolytic processing of covalent protein-linked DNA double-strand breaks.. Nature.

[34] Pouliot JJ, Yao KC, Robertson CA, Nash HA (1999). Yeast gene for a Tyr-DNA phosphodiesterase that repairs topoisomerase I complexes.. Science.

[35] Nitiss KC, Malik M, He X, White SW, Nitiss JL (2006). Tyrosyl-DNA phosphodiesterase (Tdp1) participates in the repair of Top2-mediated DNA damage.. Proc Natl Acad Sci U S A.

[36] He DM, Lee SE, Hendrickson EA (1996). Restoration of X-ray and etoposide resistance, Ku-end binding activity and V(D) J recombination to the Chinese hamster sxi-3 mutant by a hamster Ku86 cDNA.. Mutat Res.

[37] Jin S, Inoue S, Weaver DT (1998). Differential etoposide sensitivity of cells deficient in the Ku and DNA-PKcs components of the DNA-dependent protein kinase.. Carcinogenesis.

[38] Adachi N, Suzuki H, Iiizumi S, Koyama H (2003). Hypersensitivity of nonhomologous DNA end-joining mutants to VP-16 and ICRF-193: implications for the repair of topoisomerase II-mediated DNA damage.. J Biol Chem.

[39] Dedon PC, Salzberg AA, Xu J (1993). Exclusive production of bistranded DNA damage by calicheamicin.. Biochemistry.

[40] Martensson S, Nygren J, Osheroff N, Hammarsten O (2003). Activation of the DNA-Dependent Protein Kinase by Drug-Induced and Radiation-Induced DNA Strand Breaks.. Radiat Res.

[41] Elmroth K, Nygren J, Martensson S, Ismail IH, Hammarsten O (2003). Cleavage of cellular DNA by calicheamicin gamma1.. DNA Repair (Amst).

[42] Ismail IH, Nystrom S, Nygren J, Hammarsten O (2005). Activation of ataxia telangiectasia mutated by DNA strand break-inducing agents correlates closely with the number of DNA double strand breaks.. J Biol Chem.

[43] Blocher D, Kunhi M (1990). DNA double-strand break analysis by CHEF (clamped homogeneous electrical field) electrophoresis.. Int J Radiat Biol.

[44] Chen CZ, Sutherland JC (1989). Gel electrophoresis method for quantitation of gamma ray induced single- and double-strand breaks in DNA irradiated in vitro.. Electrophoresis.

[45] Wlodek D, Olive PL (1990). Physical basis for detection of DNA double-strand breaks using neutral filter elution.. Radiat Res.

[46] Tanaka T, Halicka HD, Traganos F, Seiter K, Darzynkiewicz Z (2007). Induction of ATM activation, histone H2AX phosphorylation and apoptosis by etoposide: relation to cell cycle phase.. Cell Cycle.

[47] Martensson S, N J, Osheroff N, Hammarsten O (2003). Activation of the DNA-dependent protein kinase by drug-induced and radiation-induced DNA strand break.. Radiat Res.

[48] Bowman KJ, Newell DR, Calvert AH, Curtin NJ (2001). Differential effects of the poly (ADP-ribose) polymerase (PARP) inhibitor NU1025 on topoisomerase I and II inhibitor cytotoxicity in L1210 cells in vitro.. Br J Cancer.

[49] Hsiang YH, Lihou MG, Liu LF (1989). Arrest of replication forks by drug-stabilized topoisomerase I-DNA cleavable complexes as a mechanism of cell killing by camptothecin.. Cancer Res.

[50] Ismail IH, Wadhra TI, Hammarsten O (2007). An optimized method for detecting gamma-H2AX in blood cells reveals a significant interindividual variation in the gamma-H2AX response among humans.. Nucleic Acids Res.

[51] Muslimovic A, Ismail IH, Gao Y, Hammarsten O (2008). An optimized method for measurement of gamma-H2AX in blood mononuclear and cultured cells.. Nat Protoc.

