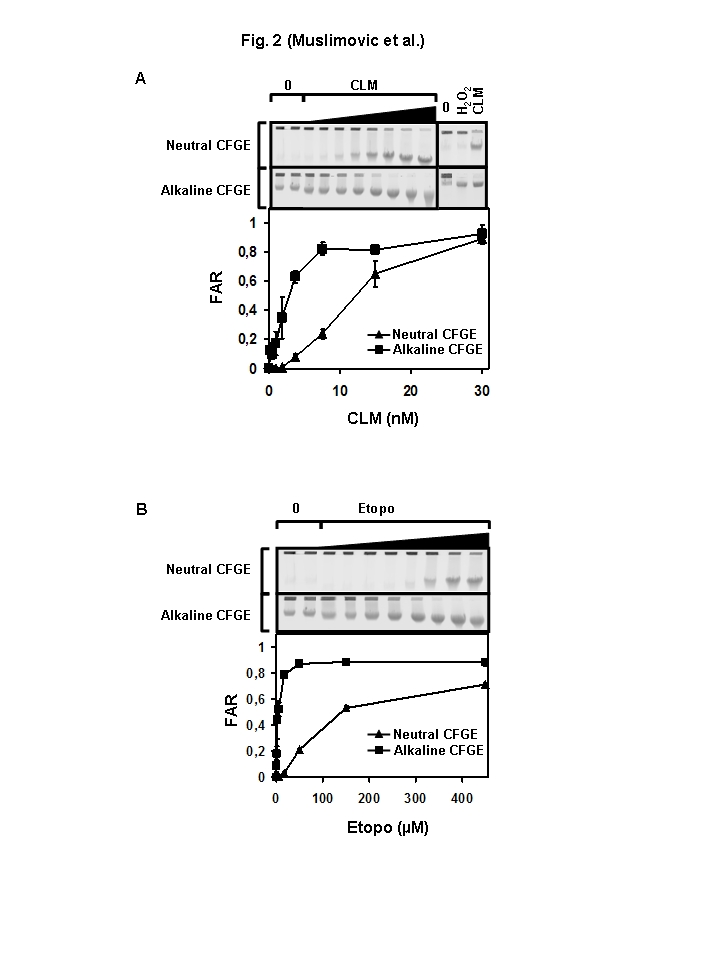# Correction: Numerical Analysis of Etoposide Induced DNA Breaks

**DOI:** 10.1371/annotation/290cebfd-d5dc-4bd2-99b4-f4cf0be6c838

**Published:** 2009-06-25

**Authors:** Aida Muslimović, Susanne Nyström, Yue Gao, Ola Hammarsten

In panels A and B of Figure 2, the legend symbols have been switched. Filled squares should represent Alkaline CFGE and filled triangles should represent Neutral CFGE. Please view the correct figure here: 

**Figure:**