# Supplementary figures and images for: Numerical Analysis of Etoposide Induced DNA Breaks
Source: PLoS One. 2009 Jun 10;4(6):e5859. doi: 10.1371/journal.pone.0005859 (PMC2689654; doi:10.1371/journal.pone.0005859)

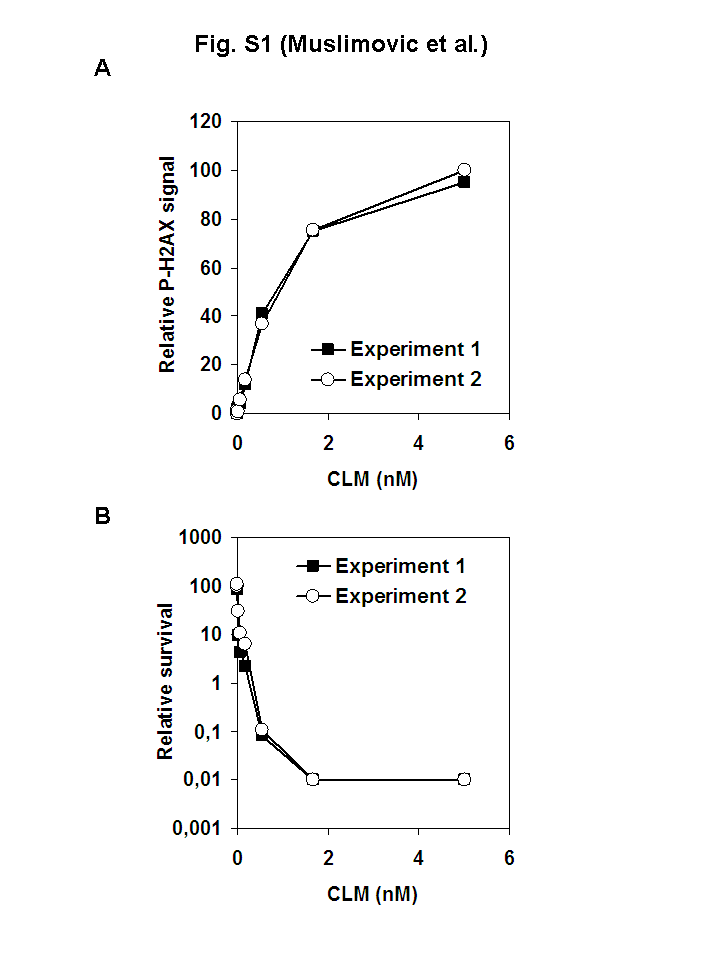

Supplement: Figure S1 — DNA strand break induction and H2AX phosphorylation by calicheamicin. SV40-transformed fibroblasts were treated with 0–5 nM CLM for 40 minutes before analysis of H2AX phosphorylation and survival using the colony assay. Data generated from different days are shown. (0.07 MB TIF) [file pone.0005859.s001.tif]

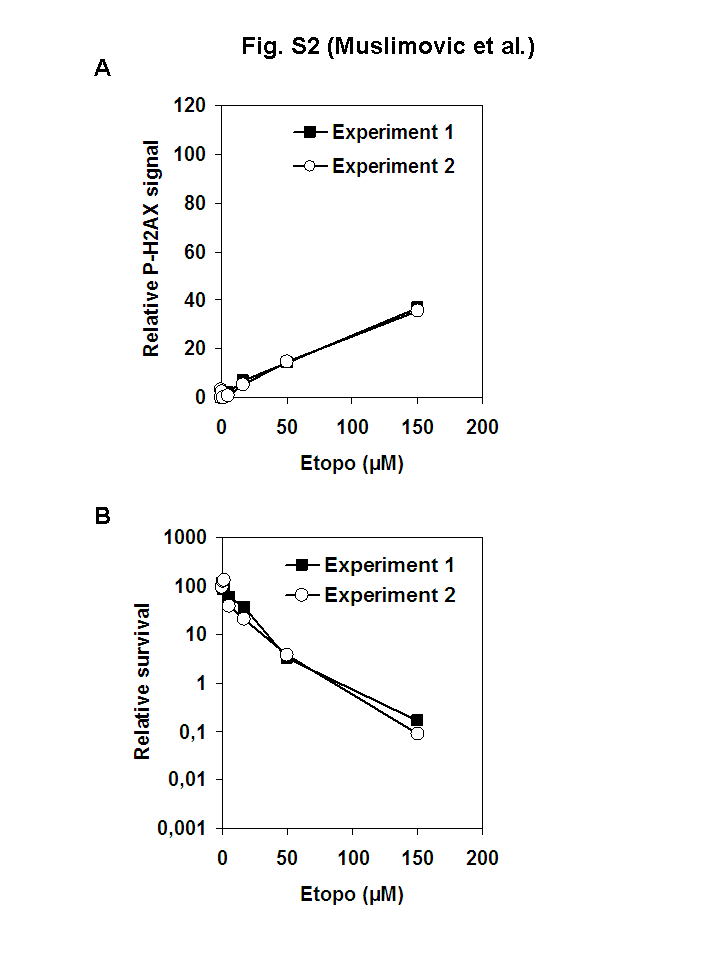

Supplement: Figure S2 — DNA strand break induction and H2AX phosphorylation by etoposide. SV40-transformed fibroblasts were treated with 0–150 µM etoposide for 40 minutes before analysis of H2AX phosphorylation and survival using the colony assay. Data generated from different days are shown. (0.07 MB TIF) [file pone.0005859.s002.tif]
